# Gastrointestinal Tuberculosis: Clinical Presentations and Diagnostic Approaches

**DOI:** 10.3390/jcm14134398

**Published:** 2025-06-20

**Authors:** Timur Gonchar, Mauro Sidney De Robertis, Carola Güther, Madlen Löbel, Tobias Kleemann

**Affiliations:** Medizinische Universität Lausitz—Carl-Thiem, 03048 Cottbus, Germany; iamtimurgonchar@gmail.com (T.G.);

**Keywords:** tuberculosis, gastrointestinal tract, Crohn’s disease

## Abstract

**Background**: Gastrointestinal tuberculosis (GI TB) is a rare form of extrapulmonary TB that often mimics other conditions, such as Crohn’s disease (CD) or GI malignancies. Conventional diagnostics, like direct microscopy and culture, are often inconclusive or slow, delaying treatment. In Germany, a low-incidence country, GI TB is underrecognized. Rising migration has led to a resurgence of TB cases, increasing the likelihood of encountering extrapulmonary presentations. This study evaluates the performance and utility of various diagnostic tools and proposes a diagnostic approach to reduce delays and avoid unnecessary interventions. **Methods**: We retrospectively analyzed eight patients suspected of GI TB based on clinical presentation and testing. Two recent cases are described in detail to highlight diagnostic and therapeutic challenges. **Results**: GI TB was confirmed in five cases (62.5%), and all the patients presented with abdominal complaints, with the majority experiencing systemic symptoms such as weight loss or fever. Histopathology supported the diagnosis in all GI TB cases, while PCR testing was positive in four. Direct microscopy detected acid-fast bacilli in only one case. The remaining patients were diagnosed with latent genital TB, disseminated TB without GI involvement, or were ruled out clinically. **Conclusions**: GI TB remains a diagnostic challenge that often mimics other conditions, such as CD or malignancy. Early use of histopathology and PCR in patients with a high risk of GI TB is critical for timely diagnosis. In low-incidence settings like Germany, clinicians should maintain high suspicion in at-risk populations (e.g., migrants from areas or immunocompromised patients), especially when symptoms mimic CD or malignancy, to improve outcomes and avoid unnecessary procedures.

## 1. Introduction

Tuberculosis (TB) is an infectious disease caused by *Mycobacterium tuberculosis*, typically spread through aerosol droplets. Two forms are recognized: primary TB, which is often asymptomatic in immunocompetent individuals, and secondary TB, an active infection that usually affects the lungs. The infection can spread hematogenously or via the lymphatic system, potentially involving multiple organs [1]. Although rare, gastrointestinal tuberculosis (GI TB) accounts for up to 3% of all TB cases [2,3,4].

The diagnosis and treatment of GI TB are challenging due to the low sensitivity of symptoms and its nonspecific clinical presentation. Clinical features include constitutional symptoms such as anorexia, weight loss, and fever, as well as abdominal manifestations, such as a change in bowel habits, gastrointestinal bleeding, or organ masses [5]. These diverse presentations have led to GI TB being referred to as the “great mimicker” [6,7]. Differentiating GI TB from Crohn’s disease (CD) can be particularly challenging due to similar symptom constellations and clinical findings [8,9]. However, treatment strategies differ significantly. Other important differential diagnoses include abdominal malignancies [10,11,12]. Given the frequent overlap in clinical and imaging findings, accurate diagnosis requires a high index of suspicion and a thorough diagnostic workup.

GI TB is more common among individuals of lower socioeconomic status, often linked to limited health literacy and malnutrition [13]. It also occurs more frequently in immunocompromised patients, including those with human immunodeficiency viruses (HIV), post-transplant patients, or individuals taking immunosuppressive medications [14,15,16,17].

Treatment for GI TB follows standard protocols for extrapulmonary TB: isoniazid, rifampicin, pyrazinamide, and ethambutol for eight weeks, followed by isoniazid and rifampicin for four months [13,18].

TB remains a globally prevalent disease, affecting approximately 20% of the world’s population [19]. Endemic regions include India, Pakistan, and China [20]. Although TB is relatively rare in Germany, incidence rates have been increasing since 2013 [21]. An important contributing factor is increased migration from regions with higher TB prevalence [22]. Due to the low number of recorded cases, research on GI TB in Germany remains limited. In this article, we aim to evaluate the diagnostic performance and utility of different diagnostic methods in GI TB. Furthermore, we seek to outline the challenges in differentiating GI TB from conditions with similar clinical presentations. Finally, we propose a diagnostic approach designed to reduce delays and prevent unnecessary interventions.

## 2. Methods

A retrospective analysis was conducted on eight patients with a high clinical suspicion of TB involving gastrointestinal or pelvic organs at the Carl-Thiem-Hospital in Cottbus, Germany, between 2003 and 2023. This observational study aims to describe the correlation between primary complaint and confirmed tuberculosis diagnosis and the diagnostic and treatment modalities chosen with the presentation on the follow-up. Data regarding primary complaints, histopathological examinations, direct microscopy for acid-fast bacilli (AFB), and polymerase chain reaction (PCR) results were extracted and analyzed.

The inclusion and exclusion criteria for the study are summarized in Table 1. Patient data were extracted retrospectively from a pre-identified patient list by reviewing medical records for relevant clinical information and diagnostic test results, specifically focusing on the investigations outlined earlier in this study. The diagnosis of GI TB was established based on a combination of clinical features with molecular (PCR) and/or histopathological findings. Histopathological confirmation required the presence of caseating granulomas and/or AFB in a biopsy specimen acquired from endoscopy or surgery. Data regarding additional diagnostic tools, such as direct microscopy, were used when available but were not required for classification as GI TB in this study.

Direct microscopy with acid-fast staining utilizes Ziehl–Neelsen stain with carbol fuchsin to detect *M. tuberculosis*. Albeit widely used, the sensitivity of this test is low, and high bacterial burden is necessary to detect the organism [23,24]. PCR is a rapid and sensitive method for detecting *Mycobacterium tuberculosis* by amplifying its genetic material, allowing for earlier diagnosis compared to traditional tests. However, in low-incidence, high-resource settings, the specificity of the test may be limited [23,25].

In addition to the retrospective analysis, we provide the case reports for two recent patients, which entail clinical presentations, diagnostic challenges encountered, and management strategies used in both cases.

The ethical approval for this study was given by the Ethics Committee of the State Chamber of Physicians of Brandenburg (Ethikkommission der Landesärztekammer Brandenburg), approval number [2025-44-BO-ff].

## 3. Results

The study cohort consisted of eight patients with varied presentations and clinical courses, all summarized in Table 2. In the table, tuberculosis test results are reported as “Positive” or “Negative” to reflect the presence or absence of infection as determined by each diagnostic method. A ‘-’ symbol indicates that a particular test was not performed on that participant.

Constitutional symptoms, defined as fever, sweating, or weight loss, were present in four out of eight patients (50%). Nonspecific gastrointestinal symptoms, such as abdominal pain, signs of ascites, melena, or gastroenteritis manifestation, affected six out of eight patients (75%) and often prompted further evaluation for gastrointestinal involvement. Two patients (25%) also reported urinary symptoms such as dysuria or incontinence, raising suspicion for peritoneal or genitourinary involvement. Pulmonary symptoms, such as cough and dyspnea, only occurred in two out of eight patients (25%). In both cases, they coexisted with either systemic symptoms and/or gastrointestinal presentation.

Histopathology revealed granulomatous inflammation consistent with tuberculosis in the majority of confirmed GI TB cases. All these patients presented with gastrointestinal symptoms, and 75% of them showed systemic signs. PCR testing showed higher diagnostic yield than direct microscopy, which was rarely positive. Among the three non-GI TB cases, one was diagnosed with latent genital tuberculosis via interferon gamma release assay (IGRA), one had disseminated TB with psoas abscess and spinal involvement (Pott’s disease), and one had no evidence of TB following clinical assessment. In this last case, detailed evaluation and GI consultation helped avoid unnecessary testing.

Given the limited sample size and retrospective observational study design, statistical comparisons and hypothesis testing were not conducted. The results are intended to provide descriptive insight into clinical presentations and diagnostic approaches in suspected gastrointestinal tuberculosis.

Two selected cases are described in detail to illustrate the clinical diversity and diagnostic challenges of GI TB.

### 3.1. Case 1

A 76-year-old female presented with persistent abdominal pain, constipation, fever, and night sweats lasting over three weeks. She was in stable general condition with normal nutritional status. Physical examination revealed a distended abdomen and lower abdominal tenderness.

**Figure 1:**
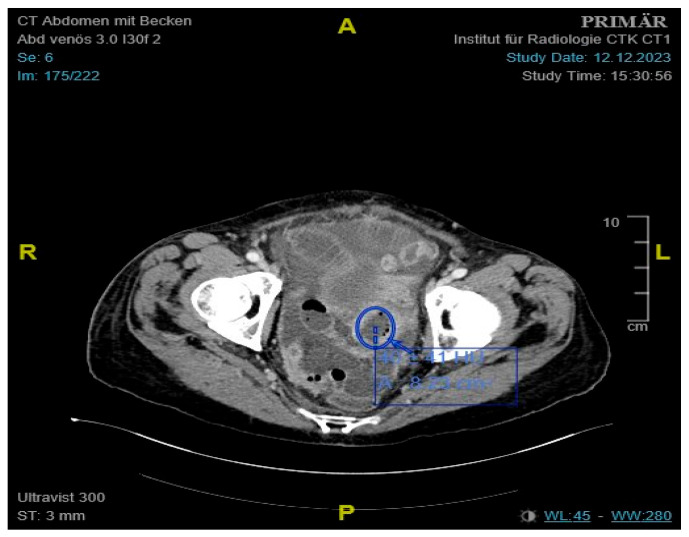
Contrast-enhanced CT of the abdomen showing an approximately 4.1 cm mass, consistent with a small bowel conglomerate tumor.

**Figure 2:**
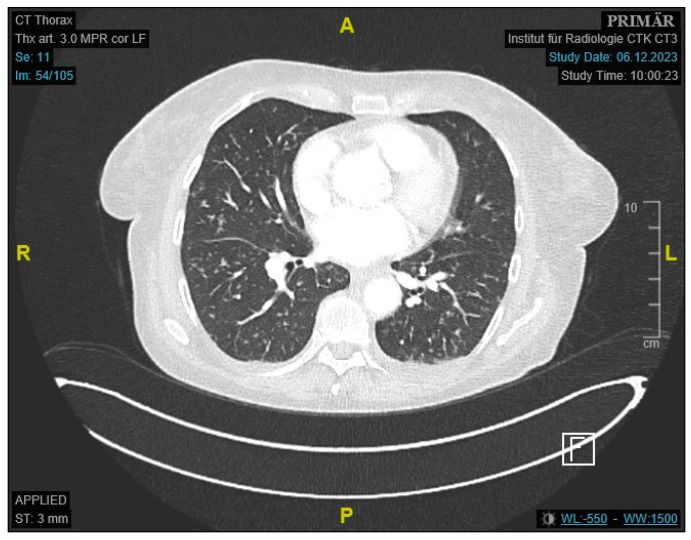
Contrast-enhanced CT of the thorax showing scattered pulmonary consolidations.

**Figure 3:**
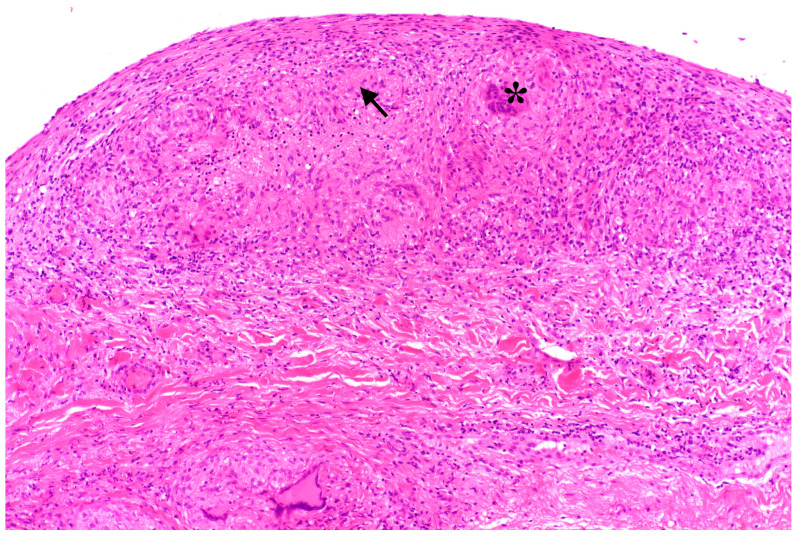
Histopathologic peritoneal sample showing subperitoneal multifocal granuloma-like changes (arrow) with epithelioid cell morphology and giant cells (marked with *).

Initial labs showed microcytic anemia with suspicion of iron deficiency. Abdominal ultrasound showed marked ascites and findings suggestive of peritoneal carcinomatosis. A CT of the abdomen revealed a small bowel conglomerate tumor (Figure 1) and enlarged paraaortic lymph nodes. Chest CT demonstrated bilateral scattered consolidations suspicious for metastatic spread (Figure 2). Bronchoscopy was unremarkable, and direct microscopy was negative for AFB. Subsequently, the patient developed a small bowel ileus, raising suspicion for a jejunal tumor. During the subsequent emergent laparoscopy, we observed a conglomeration tumor consisting of the loops of the small intestine. Emergency laparoscopy revealed a small bowel conglomerate mass, and the biopsy from the lower abdomen showed caseating granulomatous peritonitis (Figure 3). A detailed history revealed her partner had previously been diagnosed with pulmonary tuberculosis caused by *Mycobacterium chimaera*. The patient had no follow-up for TB because her partner was considered not infectious.

Subsequently, we consulted the pulmonology department for another bronchoscopy. Even though the microscopy was repeatedly negative, PCR from bronchoalveolar lavage was positive for *Mycobacterium tuberculosis* DNA, confirming disseminated pulmonary tuberculosis with gastrointestinal involvement.

Anti-tuberculosis therapy was initiated with rifampicin, isoniazid, and pyrazinamide, and later supplemented with ethambutol following an ophthalmologic consultation. Ongoing treatment and monitoring are being conducted in collaboration with pulmonology, with improvement in clinical condition noted.

### 3.2. Case 2

A 25-year-old male with a known history of Crohn’s disease presented with malaise, exertional dyspnea, and unintentional weight loss (~5 kg). He did not exhibit any signs of an acute infection. The patient has a migration background but no recent travel history or known exposure to TB. Physical examination was largely unremarkable aside from mild abdominal guarding.

**Figure 4:**
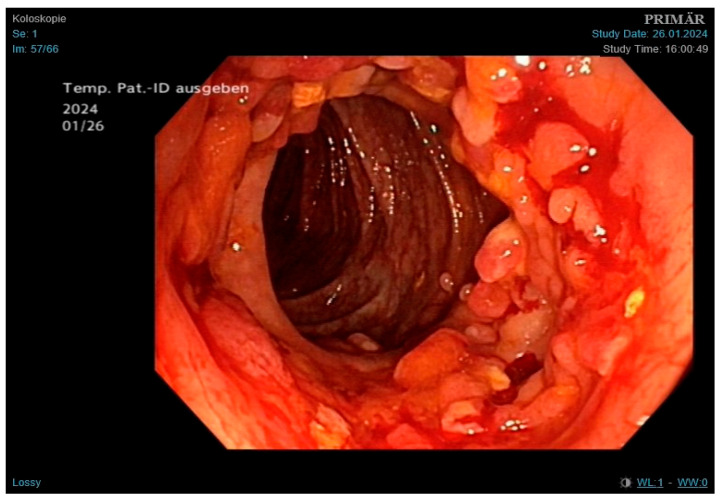
Findings obtained during colonoscopy, showing multiple pseudopolyps and fibrin-coated ulcerations; findings consistent with both Crohn’s disease and GI tuberculosis.

**Figure 5:**
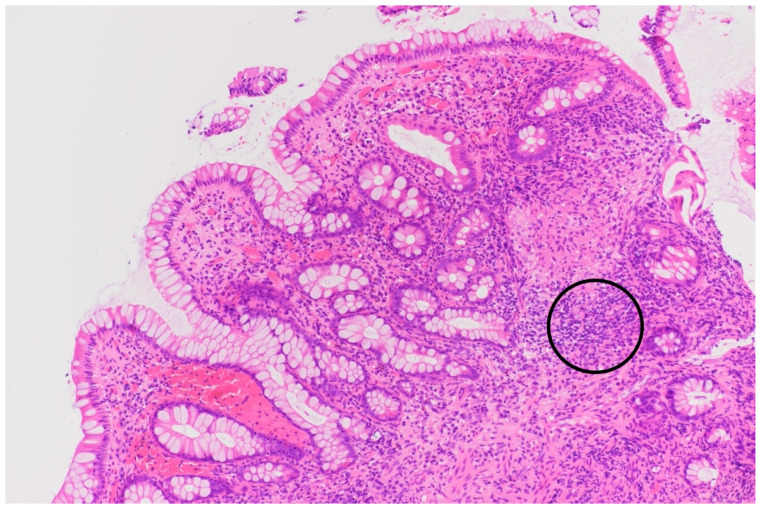
Histopathologic sample of colon showing severe inflammatory infiltrate (circle), prompting further testing to determine the cause of the colitis.

Outpatient chest X-ray showed findings suggestive of pulmonary infection. Laboratory results revealed microcytic anemia with suspected iron deficiency. Sputum microscopy and PCR confirmed *Mycobacterium tuberculosis*. Given the patient’s abdominal pain and a finding of melena in a setting of known CD, colonoscopy was performed. During the procedure, we saw numerous pseudopolyps and fibrin-coated ulcerations (Figure 4) in the cecum and colon ascendens et transversum, which are consistent with both CD and GI TB. Histopathology showed severe ulcerative and granulomatous inflammation (Figure 5) but was not able to distinguish between the two conditions. Only after subsequent PCR testing demonstrated M. tuberculosis DNA did we confirm that the active infection had caused this severe form of colitis.

The patient was diagnosed with disseminated tuberculosis affecting both the lungs and gastrointestinal tract, presenting with symptoms that mimicked a Crohn’s disease flare.

The treatment with rifampicin, isoniazid, and pyrazinamide was initiated and was later supplemented with ethambutol after an ophthalmology consult. At one-month follow-up, sputum microscopy was negative for AFB. The patient showed clinical improvement, and treatment for CD was resumed.

## 4. Discussion

The presented cases highlight the diagnostic complexity of abdominal TB in patients with nonspecific GI and systemic symptoms. In the first case, we initially suspected a gastrointestinal infection; however, imaging revealed a small bowel mass and pulmonary lesions, leading to a working diagnosis of metastatic GI malignancy. Approximately one-third of small intestinal cancers present with metastases, frequently involving the lungs [26,27]. Symptoms are often vague and may include GI bleeding and abdominal tenderness [28]. However, endoscopically obtained samples in our patient showed no malignant epithelial transformation.

The diagnostic process was further complicated by repeatedly negative bacterial cultures. On average, establishing a definitive diagnosis of tuberculosis can take up to three weeks [29]. In this case, a combination of culture, smear microscopy, and PCR was employed, with only the PCR yielding a positive result. Direct microscopy with acid-fast stain has a poor sensitivity of less than 5% for GI TB [30,31]. While bacterial culture remains the gold standard [32], it requires long incubation, up to eight weeks [33]. In our patient, the diagnosis was ultimately confirmed after a lower abdominal biopsy revealed caseating granulomas, prompting PCR testing that detected *M. tuberculosis* DNA. Although nucleic acid amplification tests offer higher sensitivity, their high cost limits their use to cases with a strong clinical suspicion [32]. The patient also showed B-symptoms, common in TB but also seen in malignancies [34]. After we had carefully taken the family medical history of the patient, we managed to find out that her partner had a past medical history of lung tuberculosis, prompting further testing. Ultimately, the diagnosis of extrapulmonary TB with GI involvement was confirmed based on clinical features, family history, and positive PCR results.

In the second case, the patient presented with imaging findings suggestive of primary TB. Both smear microscopy and PCR testing were positive, allowing for early treatment. The primary diagnostic challenge, however, was distinguishing GI TB from a CD flare. Due to overlapping features, misdiagnosis is common: up to 20% of CD patients are misdiagnosed with TB, and 10% of GI TB cases are mistaken for CD [35]. In this patient, constitutional symptoms such as malaise and weight loss favored TB, while melena pointed toward CD [9]. Histopathologic similarities in both conditions add further difficulty. During the colonoscopy, we observed multiple pseudopolyps, which can occur in both diseases. We also saw small circular ulcerations, which are more characteristic of GI TB [36]. Microscopy showed transmural inflammation and epithelioid histiocytes, also typical of both conditions. The subsequent PCR testing, however, confirmed active GI TB. This once again underlines the importance of this test in similar clinical scenarios.

Since treatment approaches for CD and GI TB differ significantly, there have been efforts to develop clinical guidelines to aid clinicians in their differentiation and management. Among these, the Asia–Pacific consensus statements are particularly important, as they address the unique challenges of unclear cases and offer practical recommendations. Since prescribing immunosuppressive medications to GI TB patients may incur serious life-threatening complications, the guideline recommends an empirical anti-TB treatment trial for 8–12 weeks if the diagnosis is uncertain [37]. Clinical monitoring and follow-up colonoscopies are recommended to assess the response to the treatment. Improved symptoms and mucosal healing implicate GI TB, and the lack of improvement suggests CD. Apart from that, it is recommended to rule out GI TB before initiating treatment for inflammatory bowel disease to avoid potential diagnosis uncertainty and medication side effects [38]. Immunosuppressive medications, such as tumor necrosis factor alpha (TNF-alpha) inhibitors or corticosteroids, can trigger further dissemination of the TB infection [39]. Administering anti-tubercular therapy to patients with CD, however, has been associated with lower relapse [40]. The evidence for such treatment is mixed and has been associated with a higher incidence of stenosis and stricture formation [41,42].

While PCR and histopathology were critical in our cohort, their cost and limited accessibility in resource-constrained settings (e.g., non-tertiary hospitals) pose challenges. We propose a tiered approach to balance diagnostic accuracy with practical constraints in low-incidence regions:

1. High suspicion (e.g., risk factors + imaging findings): Prioritize PCR/histopathology.
2. Low suspicion: Initial smear microscopy or IGRA, escalating to PCR/histopathology if clinical ambiguity persists.

**Figure 6:**
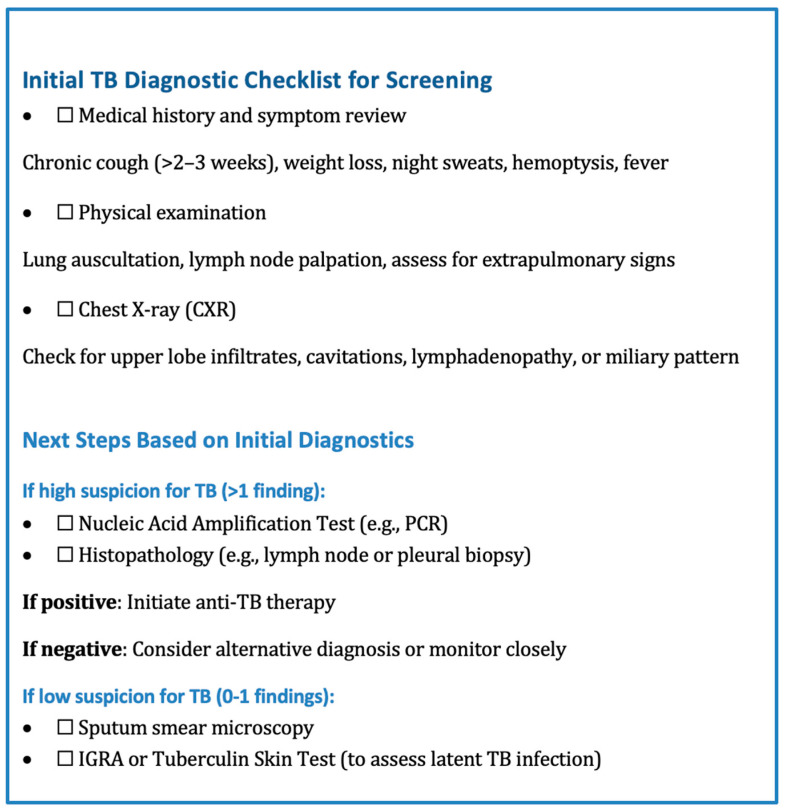
A stepwise diagnostic algorithm outlining the initial clinical assessment, imaging, and subsequent testing pathways based on findings.

Additionally, utilizing diagnostic checklists that include obtaining accurate history and reliable physical exam findings can help streamline the process (Figure 6).

Due to both the overall low prevalence of TB in Germany and the diagnostic challenges associated with the disease, recruiting enough patients for this study was challenging. This limitation contributed to the small sample size, which in turn may affect the robustness of the findings. Moreover, since more complex or atypical cases are likely to be referred to a specialized center, there is a potential for selection bias in the study population. This bias may limit the applicability of the results to the TB patient population seen in general practice. The single-center design contributes to this issue, as patient characteristics and clinical practice may differ from those in other institutions. Finally, because the study is retrospective and observational, it does not allow for causal inference and may be affected by information bias, as medical records can be incomplete or inconsistently documented.

Generally, low-incidence, high-resource countries, such as Germany, face several challenges regarding TB treatment, such as risk-group management, ensuring treatment and correct diagnosis, and reducing incidence and transmission [43]. This study is an important first step in describing GI TB in a low-incidence setting. Future research should focus on prospective multicenter studies to validate diagnostic algorithms that integrate clinical assessment and risk factors with PCR, histopathology, and other advanced diagnostic methods for earlier and more accurate identification of GI TB. An additional comprehensive cost-benefit analysis of implementing early diagnostic interventions can guide healthcare policies, optimizing patient outcomes and resource allocation.

## 5. Conclusions

The described cases demonstrate the complexity and variability of clinical presentations of TB. A thorough medical history, including family history and history of possible exposure, remains one of the most crucial aspects of the work-up. Combined with a physical exam, it helps to narrow down the list of differential diagnoses. If these measures prompt a high suspicion of TB infection, combined usage of smear microscopy and PCR testing can yield a diagnosis.

Timely diagnosis allows patients to avoid invasive measures and unnecessary treatment. Once secured, GI TB can be treated with a standard antibiotic therapy. It is important to consider such differential diagnoses as CD, since initiating false treatment may result in worse clinical outcomes.

In low-incidence settings like Germany, where GI TB is often overlooked, our study highlights the importance of the following:

1. Targeted screening in high-risk groups (e.g., migrants, immunocompromised individuals), even with nonspecific symptoms.
2. Multidisciplinary collaboration (e.g., gastroenterologists, pulmonologists, pathologists, radiologists, surgeons) to differentiate GI TB from similar conditions.
3. Prioritizing molecular diagnostics (e.g., PCR) alongside histopathology when TB is suspected, given the limitations of smear microscopy.

These strategies can mitigate diagnostic delays and optimize resource use in settings where GI TB remains rare but consequential.

In general, GI TB remains a challenging diagnosis, as patients often present with nonspecific symptoms and findings. GI TB and CD share many features and, in rare cases, may even coexist. A small intestinal mass represents an unusual but noteworthy manifestation of extrapulmonary TB, further reinforcing its reputation as a “great mimicker”. Therefore, clinicians must maintain a high index of suspicion for GI TB in patients presenting with constitutional and abdominal symptoms of unclear origin. Combining proper clinical evaluation with histopathology, AFB microscopy, and PCR testing constitutes the foundation of the workup in such cases.

In low-incidence regions, clinicians should consider GI TB in patients with:

- Chronic abdominal and constitutional symptoms (pain, weight loss) resistant to conventional therapies.
- Radiologic/histologic findings suggestive of granulomatous inflammation (e.g., ileocecal thickening, ascites).
- Risk factors such as migration from endemic areas or immunocompromised status.

Early suspicion should prompt a combination of PCR (for rapid detection) and histopathology (for granuloma confirmation), as reliance on smear microscopy alone risks missed diagnoses.

## Figures and Tables

**Table 1:** Inclusion and exclusion criteria for patients with suspected gastrointestinal tuberculosis.

Category	Criteria
Inclusion Criteria	− Age ≥ 18 years − Clinical suspicion of GI TB based on symptoms and history (e.g., abdominal pain, weight loss, fever, night sweats, diarrhea, GI bleeding) − Diagnostic testing (e.g., endoscopy, imaging, histopathology, microbiology) performed when available; absence of specific tests was not an exclusion criterion − Evaluated at Carl Thiem Hospital between January 2003 and December 2024
Exclusion Criteria	− Insufficient clinical documentation to assess symptoms and diagnostic course − Loss to follow-up before diagnostic conclusion − Workup not pursued due to patient refusal or transfer − Absence of gastrointestinal symptoms (e.g., patients evaluated solely for pulmonary TB)

**Table 2:** Summary of diagnostic findings and final diagnoses of gastrointestinal (GI) tuberculosis.

Patient	Treatment Year	Presenting Symptoms	Histopathology	Direct Microscopy (Acid-Fast Bacilli)	PCR	Final Diagnosis
1	2003	Weight loss, malaise, cough	Positive	Negative	Negative	GI Tuberculosis
2	2011	Ascites, gastroenteritis-like symptoms	Positive	-	Positive	GI Tuberculosis
3	2013	Lower abdominal pain, dysuria	Negative	-	-	Latent genital tuberculosis
4	2018	Profound sweating, lower abdominal pain	-	-	-	No TB
5	2021	Abdominal pain, lower back pain	Negative	Negative	Positive	Disseminated TB with Pott’s disease and psoas abscess
6	2021	Diffuse abdominal pain	Positive	Negative	-	GI TB, genital TB
7	2023	Exertional dyspnea, weight loss, malaise, melena	Positive	Positive	Positive	GI TB
8	2023	Abdominal pain, constipation, urinary incontinence, night sweating, subfebrile fever	Positive	Negative	Positive	GI TB

## Data Availability

The original contributions presented in the study are included in the article, further inquiries can be directed to the corresponding authors.

## Abbreviations

TB	Tuberculosis
GI TB	Gastrointestinal tuberculosis
CD	Crohn’s disease
HIV	Human immunodeficiency virus
AFB	Acid-fast bacilli
PCR	Polymerase chain reaction
IGRA	Interferon-gamma release assay
TNF-alpha	Tumor necrosis factor-alpha
